# *In-situ* hearing threshold estimation using Gaussian process classification

**DOI:** 10.1038/s41598-023-40495-w

**Published:** 2023-09-06

**Authors:** Christopher Boven, Reagan Roberts, Jeff Biggus, Malini Patel, Akihiro J. Matsuoka, Claus-Peter Richter

**Affiliations:** 1 Soundwave Hearing, LLC, 619 Enterprise Drive #205, Oakbrook, IL 60523 USA; 2 Northwestern Medical Group, 675 N. St. Clair, Suite 15-200, Chicago, IL 60611 USA; 3 Department of Otolaryngology, Northwestern University, 320 E. Superior Street, Chicago, IL 60611 USA; 4 Roxelyn and Richard Pepper Department of Communication Sciences and Disorders, School of Communication, Northwestern University, Evanston, IL 60201 USA; 5 The Hugh Knowles Center for Clinical and Basic Science in Hearing and Its Disorders, Evanston, IL 60201 USA; 6 Center for Advanced Regenerative Engineering, Evanston, IL 60201 USA; 7 Department of Biomedical Engineering, Northwestern University, 320 E. Superior Street, Chicago, IL 60611 USA; 8 Department of Otolaryngology, Northwestern University Feinberg School of Medicine, Searle Building 12-470; 303 E. Chicago Avenue, Chicago, IL 60611-3008 USA

**Keywords:** Medical research, Translational research

## Abstract

One in six Americans suffers from hearing loss. While treatment with amplification is possible for many, the acceptance rate of hearing aids is low. Poor device fitting is one of the reasons. The hearing aid fitting starts with a detailed hearing assessment by a trained audiologist in a sound-controlled environment, using standard equipment. The hearing aid is adjusted step-by-step, following well-described procedures based on the audiogram. However, for many patients in rural settings, considerable travel time to a hearing center discourages them from receiving a hearing test and treatment. We hypothesize that hearing assessment with the patient’s hearing aid can reliably substitute the hearing test in the clinic. Over-the-counter hearing aids could be programmed from a distance and fine-tuned by the hearing aid wearer. This study shows that a patient-controlled hearing assessment via a hearing aid in a non-clinical setting is not statistically different from an audiologist-controlled hearing assessment in a clinical setting. The differences in hearing obtained with our device and the Gaussian Process are within 3 dB of the standard audiogram. At 250 Hz, the sound delivery with the hearing aid used in this study added an additional reduction of sound level, which was not compensated.

## Introduction

Hearing loss is the third most common chronic medical condition in the United States, affecting over 30 million Americans, including half of the adults between 70 and 79 years old^1–3^. Estimates show that less than 20% of these individuals wear hearing aids^4–6^. Several factors lead to the high incidence of untreated hearing loss, including the high cost of devices and limited access to professional services^7, 8^. Previous research has shown that untreated hearing loss correlates with accelerated cognitive decline, resulting in dementia, anxiety, and depression^9–16^. To better treat deaf or hard-of-hearing individuals, the government has removed some regulations for distributing hearing aids^17^. This regulatory change will ease patient access to hearing aids. However, it will also create challenges for hearing aid providers because the devices should be programmable outside of a clinical setting, at the patients’ homes, for example, through telemedicine. Enabling hearing aid access may also increase the patients’ acceptance and use of the devices. The proposed changes for over-the-counter hearing aids also require changing the standard processes for self-fitting^16, 18^. This clinical study is an effort to provide a method for patients to adjust their hearing aids outside of a clinical setting using their own hearing aids. Virtually all modern hearing aids are fitted using the patient’s audiogram, determined by pure tone audiometry in a controlled clinical setting, by trained personnel, using instruments specifically designed for this purpose^19–22^. The audiologist determines the patient’s hearing by incrementally approximating the lowest intensity for a pure tone that can still be perceived at a set of standard frequencies ranging from 125 Hz to 8 kHz^23^. At each frequency, a staircase “up 5 dB—down 10 dB” approach is used^24^. It takes an audiologist 10–15 min to complete an audiogram. Hearing aid amplification settings can be assigned using well-known prescriptive standards such as National Acoustic Laboratories (NAL), Desired Sensation Level (DSL), or others using the patient's audiogram^25, 26^. Despite pure-tone audiometry is an indispensable component of the hearing aid fitting process, eminent limitations of standard audiometry exist:

1. A trained professional must administer a hearing test with specialized equipment, including a sound booth.
2. A statistical estimate of confidence in the result is generally not available with standard audiometry.
3. Standard test procedures do not use all information available in the data by assuming that the hearing thresholds at different frequencies are uncorrelated, leading to redundant measurements to achieve the desired accuracy and longer testing times.
4. Restricting the stimuli to a fixed set of standard frequencies requires additional assumptions to estimate the threshold at other frequencies, particularly when fitting hearing aids with a large number of channels.

We test a novel approach with our clinical study, addressing the above limitations. The new procedure combines *in-situ* pure-tone audiometry with Bayesian statistical inference. With this, we name “*In-situ* hearing testing” as the use of hearing aids to administer a hearing test rather than specialized audiometry equipment. This approach uses a Gaussian Process Classification^27, 28^ to obtain continuous pure-tone threshold curves in less time than a traditional audiogram. The Gaussian Process Classification also provides a measure of confidence in its threshold estimates and requires only the hearing aids and a connected smartphone to conduct. In recent years, *in-situ* audiometry and Bayesian hearing tests have been separately compared to conventional pure-tone threshold testing, producing results comparable to a standard audiogram^28–33^. However, the threshold was tested using the same standard equipment in the clinic to assess hearing. A combined approach that uses the person’s hearing aids and probabilistic models to optimize *in-situ* hearing tests has not yet been explored.

Note that the audiogram is the starting point for the fitting process, and the need for alignment between the audiogram obtained by the audiologist or obtained by the self-fitting procedure is primarily to satisfy the need for documentation. Following this logic, no audiogram would be required for the fitting process. The fitting could start with an arbitrary audiogram, aligning somewhat with the patient's hearing ability. Using such an approach, the fitting will likely take more iterations. We suggest that a good alignment of the results from the self-fit will optimize the fitting procedure.

## Results

### Test subjects

Patients who had an appointment with the hearing clinic for a hearing assessment and had no severe-to-profound hearing loss were invited to participate in the study. Driven by patient availability, we did not control gender, ethnicity, and race. Among the participants, 37 (70%) were females, and 16 (30%) were males (Table 1). The age of the participants was similar for both genders. For females and males combined, the study participants’ age ranged from 24 to 81 years, with a median of 54 years, an average age of 51.3 years, a standard deviation of 16.6 years, and a 95% confidence interval between 46.9 and 55.8 years (Table 1). The female age ranged from 24 to 81 years, with a median of 54 years, an average age of 53.3 years with a standard deviation of 17.9 years, and a 95% confidence interval between 47.5 and 59.1 years (Table 1). The age of the males ranged from 25 to 81 years, with a median of 55 years, an average age of 53.9 years with a standard deviation of 17.9 years, and a 95% confidence interval between 45.1 and 62.7 years (Table 1). Using the t-test provided with the IGOR Pro8 statistical package, age differences between females and males, mean-age_females_ ! = mean-age_males_, were shown not to be significantly different (alpha = 0.05, the critical value t__stat_ = 1.5, n_females_ = 37, n_males_ = 16, DF_females_ = 36, DF_males_ = 15, P = 0.132).

**Table 1:** The counts (N) of the female and male participants and their ages are shown.

	N	%	Age (years)					
			Mean	Stdev	95% CI	Min	Median	Max
Females	37	70%	53.3	17.9	(47.5, 59.1)	24	54	81
Males	16	30%	46.8	12.1	(40.9, 52.7)	25	55	81
Both gender	53	100%	51.3	16.6	(46.9, 55.8)	24	54	81

*CI* confidence interval, *stdev* standard deviation.

### Audiograms

**Figure 1:**
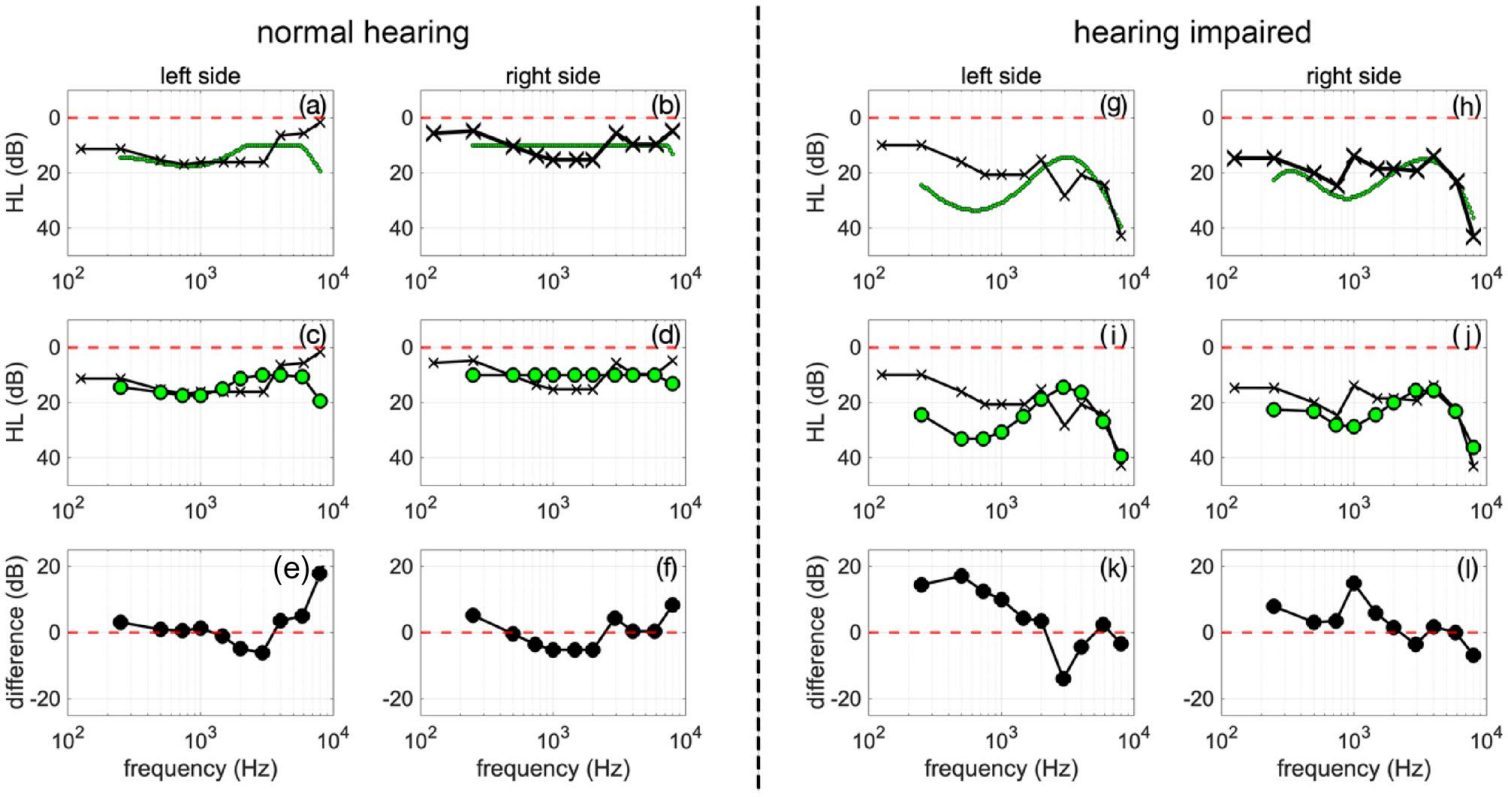
The figure shows audiograms obtained from two study participants, one with normal hearing (panel (**a**) through (**f**)) and one with hearing loss (panel (**h**) through (**l**)). The traces in the top row with the “x” markers show the behavioral hearing thresholds at discrete frequencies, at which hearing was assessed during standard audiometry by a certified audiologist in a sound-reduced chamber with standard equipment. The green traces in the top row with the “o” markers show pure tone thresholds of the same patient obtained with a self-guided *in-situ* testing procedure using hearing aids in a sound-reduced environment. The second row shows the thresholds for the different methods at the same frequencies. The third row shows the difference between the methods. Both methods provided similar results for the hearing thresholds of the patients. The broken red line indicates the zero line or normal hearing.

Figure 1 shows the standard audiogram (method M1), plotted along with the behavioral hearing threshold obtained “*in-situ*” with a hearing aid and the Gaussian Process classifier (method M2). While the standard audiogram collected values at 11 distinct frequencies: 125, 250, 500, 750, 1000, 1500, 2000, 3000, 4000, 6000, and 8000 Hz (Fig. 1a and b), hearing assessment with the Gaussian Process classifier provided data at many more frequencies. To compare the audiograms captured with the two methods, corresponding points to the standard audiogram are selected from the audiogram obtained with M2 (Fig. 1). The audiograms are shown in Fig. 1a to j for the left and right sides, respectively. The differences between the two audiograms obtained with M1 and M2 are shown in Figs. 1e,f,k and l. Data is shown for two subjects, one with normal hearing and the other with apparent high-frequency hearing loss.

**Figure 2:**
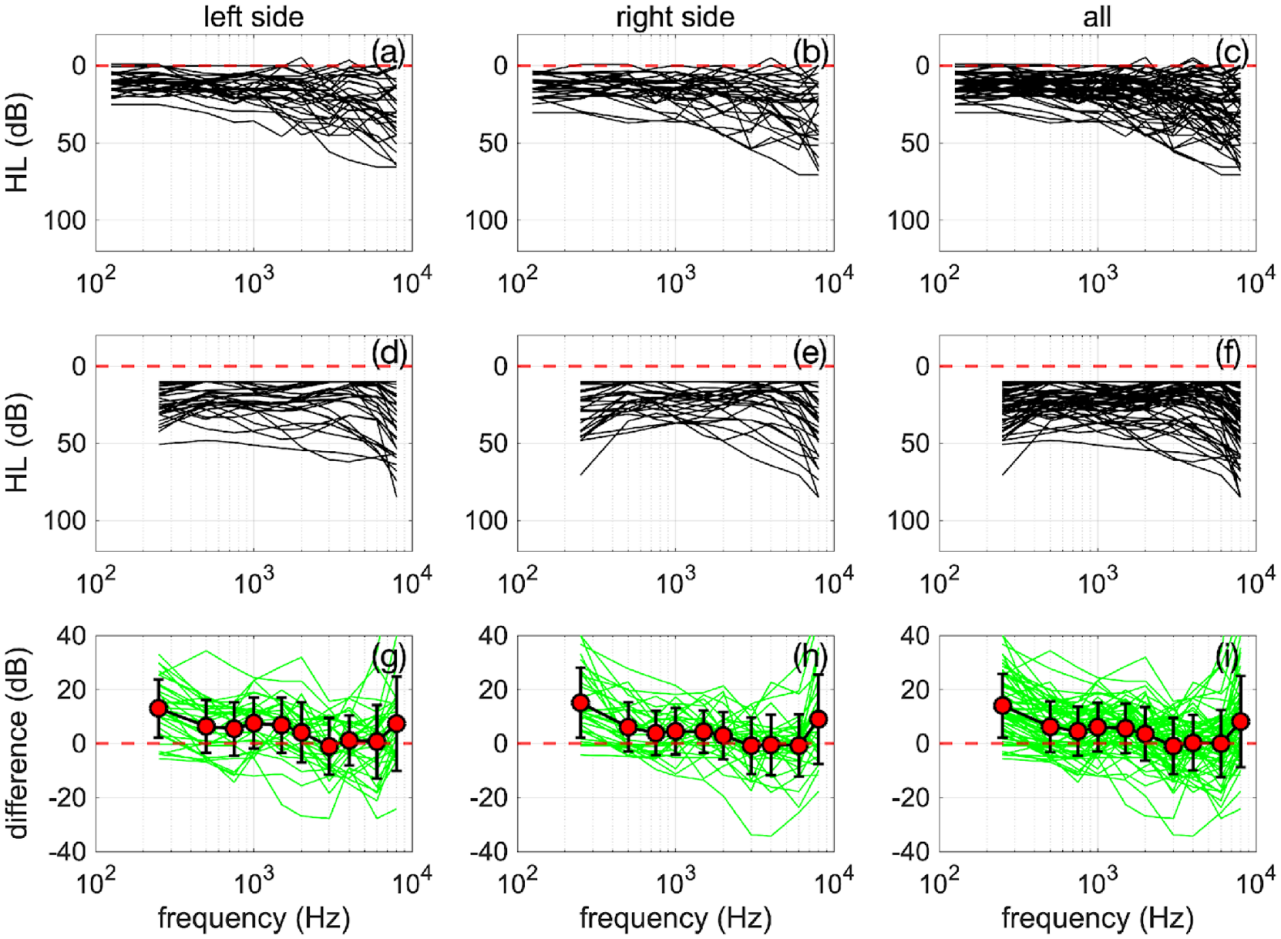
The panels show plots of all audiograms captured with M1 and M2 in a study participant's left and right ear. In panels (**a**–**c**), the results for M1 at the standard frequencies are shown. Panels (**d–f**) show the corresponding results obtained with M2. Panels (**g–i**) provide the difference between the results obtained with M1 and M2. The green lines are the individual difference; the red circles show the average ± one standard deviation. The broken red line indicates the zero line.

The cumulative results for all study participants are shown in Table 2 and Fig. 2.

**Table 2:** The table shows the results for the average pure tone hearing levels on the left (HL_L) and right (HL_R) obtained through standard audiometry (M1) and our procedure (M2) using the hearing aids and the GP for the left side (GP_L) and the right side (GP_R).

Dataset	HL_L	HL_R	GP_L	GP_R	diff_L	diff_R
1	27.2	26.8	31.5	32.3	4.3	5.4
2	15.6	17.1	11.5	17.0	−4.1	−0.1
3	16.8	16.4	24.7	24.8	7.9	8.4
4	26.3	18.1	34.4	12.9	8.2	−5.2
5	19.8	16.2	12.7	20.8	−7.1	4.6
6	19.2	25.9	16.5	43.0	−2.6	17.1
7	15.2	13.7	9.2	18.0	−6	4.3
8	6.6	9.4	14.0	11.3	7.4	1.9
9	29.3	29.3	38.5	26.5	9.2	−2.8
10	28.0	27.7	35.8	32.0	7.8	4.3
11	3.4	0.5	11.3	11.2	7.9	10.7
12	13.7	9.8	18.8	10.2	5	0.4
13	8.8	19.3	18.6	14.4	9.9	−5
14	5.7	8.8	14.6	9.6	8.9	0.7
15	29.9	29.8	30.2	19.7	0.3	−10.1
16	16.1	16.8	21.9	13.0	5.8	−3.8
17	15.0	19.3	15.6	38.9	0.7	19.6
18	28.8	32.3	32.1	37.1	3.3	4.8
19	12.1	10.0	12.9	9.4	0.8	−0.6
20	2.8	6.6	22.3	10.1	19.5	3.6
21	7.2	5.4	16.3	10.2	9.1	4.7
22	44.0	46.1	49.4	48.9	5.4	2.8
23	3.5	4.1	10.7	12.2	7.2	8.1
24	8.5	9.5	20.3	11.1	11.8	1.6
25	22.0	17.7	18.4	23.8	−3.5	6.1
26	19.7	28.6	12.4	11.3	−7.3	−17.4
27	12.3	10.6	12.4	29.8	0.2	19.2
28	16.1	14.9	13.2	19.8	−2.8	4.9
29	16.9	16.3	26.1	13.4	9.2	−2.8
30	16.3	12.7	10.5	20.1	−5.8	7.4
31	22.3	35.9	10.0	22.7	−12.3	−13.2
32	21.2	20.7	40.3	33.6	19.1	12.9
33	23.2	22.6	30.6	25.4	7.4	2.8
34	6.5	6.4	11.7	9.2	5.2	2.7
35	20.9	20.4	23.8	21.6	3	1.2
Count	35	35	35	35	35	35
Averages	17.2	17.9	20.9	20.7	3.8	2.8
Stdev	9.17	9.96	10.31	10.68	7.16	7.92
Serr	1.55	1.68	1.74	1.81	1.21	1.34
Avg+tc*serr	20.2	21.2	24.4	24.2	6.2	5.5
Avg−tc*serr	14.1	14.6	17.5	17.2	1.4	0.2
Tc = 1.96						

Differences between the HL-L and GP_L, and HL_R and GP_R are calculated separately. At the bottom of the table, the number of data sets (count), the averages, standard deviations (stdev), standard error (serr), and confidence intervals are calculated CI 95%[avg + tc*serr, avg + tc*serr], where *avg* denotes the averages, *tc* the critical statistical value, and *serr* the standard error.

In Fig. 2, the left column shows the results from the left ears, the middle columns from the right ears, and the right column is the cumulative data from the left and right ears. The top row shows the standard audiograms from the study’s participants, with normal hearing to mild hearing loss. The second row shows the corresponding audiograms obtained with M2. The third row shows the differences for each audiogram determined by the two methods in green. The circles show the averages, and error bars indicate the standard deviation.

The average difference ± one standard deviation between the M1 and M2 threshold estimate across all subjects and frequencies was 3.8 ± 7.2 (N = 35) on the right and 2.8 ± 7.9 dB (N = 35) on the left side. They are close to zero at many frequencies, but lower frequencies tend to deviate more. It should be noted that while audiogram values collected from M1 could take on values below 10 dB HL, for statistical analysis, M1 values were limited to 10 dB HL to match the dynamic range of M2.

**Figure 3:**
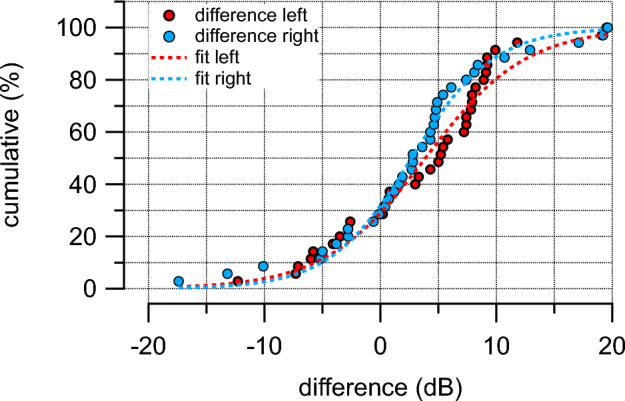
The figure shows the differences for all data sets as a cumulative plot. The 50% difference is shifted to the right by 3.8 ± 7.2 (N = 35) on the right and 2.8 ± 7.9 dB (N = 35) on the left side.

The distribution of the differences in the pure tone averages is shown in Fig. 3. The plot is a cumulative plot showing that 80% of the data obtained are within a range of ± 10 dB.

### Statistical evaluation

#### Testing the pure tone threshold average differences for a mean equal to zero

The difference between the pure tone threshold averages (PTAs), obtained with M1 and M2, are normally distributed with an unknown mean and variance. The sample data was tested for matching a normal distribution using the Lilliefors test. The test is available in MATLAB, h = *lillietest*(x), where ‘h’ is the test decision for the hypothesis that the data ‘x’ come from a normal distribution with an unknown mean and variance (0 = true; 1 = false), at the 5% significance level. For our data, the test was h = 0 for the data from the right or left side. The t-test for a mean equal to zero and alpha = 0.05 in MATLAB, [h,p,ci,stats] = *ttest*(x), has the following results on the right side: h = 0, the probability p = 0.41; the confidence interval ci = (− 0.82, 6.5), and the test statistics, ‘stats’, produced a value for the statistics tstat = 2.12, with the degree of freedom df = 34, and an estimated population standard deviation sd = 7.9. The returned value h = 0 indicates that the t-test accepts the null hypothesis that the sample data comes from a population with a mean equal to zero at the 1% significance level.

For the left side, the values are: h = 1, the probability p = 0.004; the confidence interval ci = (0.49, 7.1), and the test statistics, ‘stats’, produced a value for the statistics tstat = 3.13, with the degree of freedom df = 34, and an estimated population standard deviation sd = 7.17. The returned value h = 1 indicates that the t-test rejects the null hypothesis that the sample data comes from a population with a mean equal to zero at the 1% significance level.

#### Comparing the audiograms with a multivariate analysis of variance (MANOVA)

Pure tone thresholds at ten different frequencies define the audiogram. Changes in the audiograms then show deviations from normal hearing. A multivariate version of the analysis of variance is therefore essential to determine whether the entire set of means of thresholds is different from one group to the next. Dependencies can be seen in the grouped plot matrix (Supplemental Figs. 1 and 2) generated with the MATLAB command *gplotmatrix*(x,[],group); ‘x’ denotes the groups, and ‘group’ the grouping variable. The MANOVA was performed with the MATLAB command [d,p,stats] = *manova1*(x,group); ‘x’ denotes the groups, ‘group’ the grouping variable, ‘d’ is an estimate of the dimension of the space containing the group means, ‘p’ is a vector of *p*-values testing whether the means lie in a space of dimension 0, 1, and so on. The command also returns ‘stats’, a structure with additional MANOVA results. The largest possible dimension is either the dimension of the space or one less than the number of groups. If the *i-*th *p*-value is near zero, it indicates that the group means do not lie on a space of *i*-1 dimensions. The choice of a critical *p*-value is specified by the value of the input argument alpha (0.05 or 0.01). For our data, *p* = 2.26*10^–6^ for the data from the left and p = 3.0*10^–12^ for the right side; the estimated dimension is 1 for each side, indicating that the data align along a line and are statistically different.

#### Credible interval analysis

A credible interval analysis was performed where the interval of interest was defined as the average difference at each frequency + /- 10 dB. The proportion of the probability distribution within the interval, assuming normality, was computed by integrating the posterior probability estimate between the interval values. This provided an estimate for the probability of future samples expected to be within 10 dB HL of the audiogram frequency.

No assumptions were made about the distribution over different values, so an uninformative prior distribution was used. Supplemental Fig. 3 shows an example of a credible interval being calculated. Supplemental Table 1 outlines the credible intervals for 10 different frequency values 250, 500, 750, 1000, 1500, 2000, 3000, 4000, 6000, and 8000 Hz.

### Power calculations

#### Sample size and data sets not included in the analysis

The target enrollment was 55 participants. Between 2018 and 06-26 and 2018-10-25, 55 test participants/patients were enrolled. The standard audiograms for three subjects obtained with M1 were not available at the analysis time. Fourteen results using M2 were not available and could not be analyzed at the analysis time. A total of 17 data sets were excluded from the data analysis for these reasons. Three data sets acquired with M2 could not be used because the results suggested a significant error in the test procedure. For data analysis, we considered 35, with the power of the study being approximately 90%.

## Discussion

With the advent of over the counter hearing aids, and the aim to better serve patients needing amplification devices, it becomes necessary to fit those devices to their needs^18, 34–40^. This study validates the Gaussian Process classification as a robust procedure for assessing hearing with a hearing aid for in-situ fitting. The hearing threshold curves collected from M1 and M2 for all analyzed subjects divided between left and right ears showed a conventional pattern. Most subjects with hearing loss displayed sloping high-frequency hearing loss. It is visible in these curves that M2 may provide higher threshold estimates in the lower frequencies, which could be related to the fact that M2 was conducted outside of a sound booth. Note that no correction has been implemented to compensate for potential reductions in sound pressure levels originating from the transducer in the hearing aid at frequencies below 500 Hz. The calibration was accepted as provided by the hearing manufacturer, Soundworld.

When computing the difference between M1 and M2 curves across all subjects, the results suggest that the experimental *in-situ* procedure is similar to the standard audiometric method, where the average difference was 3.8 ± 7.2 and 2.8 ± 7.9 dB on the right and left side, respectively. These numbers represent an average across the ten frequencies where a difference value was obtained for each subject. When looking at the difference values separated across frequency, the average differences were minor at most frequencies, although deviations occurred more frequently at frequencies below 500 Hz and above 6 kHz. It is suggested that the discrepancy in the lower frequencies may be due to the difference in the testing environment between M1 and M2. It can be argued that 3.8 ± 7.2 on the right side and 2.8 ± 7.9 dB on the left side are small amounts considering test–retest variability for a traditional audiogram can be higher than this value^41^. Our data are not as good as published results on the difference between automated and manual audiometry. Swanepoel et al.^42^ showed that hearing thresholds obtained with automated and manual audiometry were within 5 dB or less in 87% of the normal hearing and 97% in the hearing-impaired group. Their largest overall average absolute difference across frequencies was 3.6–3.9 dB for the normal-hearing group and 3.3–2.4 for the hearing-impaired group. We must emphasize that the differences in outcomes likely originate from the methods; while our study is an in-situ study and compares audiograms obtained in the booth with standard equipment and the subjects hearing aids outside a sound-reduced hearing enclosure, Swanepoel compared the audiologist driven and the self-fitting with the same equipment bot acquired in a sound reduced chamber.

With the assumption that the audiogram is the starting point for the fitting process, any starting point could be selected, and the alignment of the audiogram, which was obtained by a certified audiologist or a self-fitting procedure, is of secondary nature. The answer we will not be able to provide from our results is how well and how fast the fitting procedure will converge and whether differences in the quality of the fits will exist. Further tests are required to address the question. The audiogram is necessary for the documentation of hearing loss for insurance purposes. It is not clear whether an audiogram obtained through a self-fitting procedure that differs by more than 10 dB from the audiogram of the same subject obtained by an audiologist must be considered as not aligned.

The returned value h = 1 from the results indicates that the t-test rejects the null hypothesis that the sample data comes from a population with a mean equal to zero at the 5% significance level. Using the average difference (3.8 ± 7.2 dB on the right side and 2.8 ± 7.9 dB on the left side) as calibration, the results of M2 can be shifted by this amount as a calibration factor. The t-test of the adjusted data shows that h = 0, indicating that the data comes from a population with a mean equal to zero at the 5% significance level. In other words, with a modest scalar calibration, the sample data representing the difference between M1 and M2 can be said to come from a population with a mean equal to zero, indicating that the M1 and M2 are similar.

The output d estimates the dimension of the group means. d = 1 indicates that the means differ but fall along a line. The two most-left columns and the top two rows of SFigures 1 and 2, corresponding to 250 and 500 Hz, show differences. These data indicate that the lower frequency differences, compared to the control responses in the standard audiometric method, appear more pronounced, confirming what the difference curves in Fig. 2g–i suggest.

The credible interval analysis assumed an interval between + /− 10 dB when estimating the probability distribution of the difference values between M1 and M2 using an uninformative prior. This analysis suggests that most subjects’ M2 measurements are within 10 dB of the M1 measurement.

Previous studies suggested that hearing thresholds obtained *in-situ* with hearing aids are equivalent to currently accepted audiometric procedures^43–45^. Our results agree with those studies. DiGiovanni and Pratt reported significant differences between hearing thresholds obtained with standard and in-situ procedures^38^. It is not clear whether the differences of 6–9 dB at 500, 1000, and 2000 Hz are caused by differences in sound level produced by the transducers.

This study is not the first report on combining probabilistic modeling and information gain. Özdamar et al.^46^ published their results on classifying audiograms by sequential testing using a dynamic Bayesian approach. The procedure included the clinician’s a priori knowledge to efficiently select the next intensity and frequency. Selections were based on a probability table and the results from previous trials. More recent work has shown that a probabilistic model of the response-generating process can provide this information. Hearing threshold estimation becomes a Bayesian posterior inference using the probabilistic modeling approach. Intensity and frequency are sequentially selected by maximizing the information gained during each iteration^46^, in particular using Gaussian Process models^28, 33, 47–53^.

In his 1998 paper, Neal strongly advocated using the Gaussian Process for regression analysis and classification^54^. He argued that the Gaussian Process is the most straightforward method of defining Bayesian regression and classification models. The strengths of the models include the variety of possible covariance functions to select from. For audiometry, the most favorable kernel is the squared-exponential covariance kernel over linear or periodic kernels because it best represents the data sets. Other strengths of the Gaussian Process relate to the efficiency in data sampling^55^, the fact that the Gaussian Process provides a posterior distribution of the model’s belief of the underlying function value for a given test point. The model is a two-dimensional binary classifier in the frequency and by a psychometric function in the intensity dimension, which provides uncertainty bands on the resulting threshold estimate. The model’s output provides objective stopping criteria and allows for optimizing the next intensity and frequency selection, which is based on the previous data collected.

## Conclusions

The present study measured the similarity between the hearing threshold estimates provided by a standard audiogram procedure versus an experimental *in-situ* hearing threshold assessment. The average difference between the threshold estimates provided by these two methods was 3.8 ± 7.2 on the right side and 2.8 ± 7.9 dB on the left side. For the fitting procedure, the method provides a reasonable starting point for the actual gain finally preferred and used by the patient. The in-situ method has advantages because the systems used for testing are the same as those for delivery.

Summary of Experimental *In-Situ* Procedure:

1. It is designed to be self-administered only using wireless-enabled hearing aids and a smartphone.
2. It uses a probabilistic model to estimate the uncertainty in the estimate, which may be used to determine if a test is valid or should be retaken.
3. It uses probability theory to optimize which tones are presented to the subject using prior response information.
4. Output is a smooth, continuous curve representing the threshold estimate for each as a function of frequency.

## Materials and methods

### Ethics declaration

All experimental procedures with human subjects followed the institutional research committee's ethical standards and the 1964 Helsinki Declaration and its later amendments. The Institutional Review Boards at Northwestern University Feinberg School of Medicine reviewed and approved all procedures (STU00205797). The subjects gave informed consent before participating in this study.

### Summary description

This study collected hearing threshold curves from the left and right ears in volunteers using two methods for each subject. Method 1 (M1) was a standard pure tone audiogram, which measured the subject’s hearing sensitivity to acoustic tones at different frequencies. The assessment focused on pure-tone air conduction thresholds inside a sound booth with a certified audiologist of the hearing clinic at Northwestern Memorial Hospital (NMH). The audiologist conducted the test in a sound booth and is part of the clinical assessment for which the patient came to the clinic. Each ear was tested separately using various transducers such as headphones or inserted earphones. As it is a behavioral test, its outcome depends on the individual's response. To estimate a person's pure tone hearing threshold, the sound level of the pure tone with a given frequency was changed incrementally until the lowest level was determined that could still be perceived. A set of standard frequencies ranging from 0.125 to 8 kHz was used, and the sound level was modified before each presentation of a short tone stimulus using a staircase “up 5 dB—down 10 dB” approach^24, 56^. Sound levels start at soft, which is barely or non-audible. After assessing their hearing with a standard audiogram, patients who fulfilled the inclusion criteria were offered to participate in the study. The audiologist immediately connected interested patients with this study's designated investigator, as described in the IRB (STU00205797).

Method 2 (M2) estimated pure tone thresholds with a self-guided *in-situ* testing procedure, where wireless-enabled hearing aids (HD100, Soundworld, Oakbrook, IL, USA) connected to a mobile app on a handheld device (Apple iPhone 8 Plus smartphone) played pure tones. The mobile app contains software that can run a Gaussian Process classification algorithm to determine the pure tones and their corresponding intensities to play throughout the test. The algorithm then returns and stores a probabilistic estimate of the hearing threshold for the left and right ears. Sound levels were between 10 and 80 dB HL. After each presentation of the pure tone, the subject was asked to press a button on the screen of an iPhone, indicating on which side the tone was heard. The software then determined the subsequent frequency and sound level based on the previously recorded responses. The following tones and corresponding sound levels were sent to the hearing aid via Bluetooth, playing the pure tone at the given sound level. The results were stored locally on the iPhone. The data were erased from the iPhone after transferring the de-identified data to a secured server at Northwestern University. The hearing aid limited the maximum possible sound levels of the hearing aid. The software used here could not override the hearing aid’s safety limits.

### Hearing aids

The hearing aid was the HD100 from Soundworld. Our custom-written software sets the pure tone frequencies and their corresponding sound levels via a Bluetooth connection between an Apple iPhone 8 Plus smartphone and the hearing aid during the hearing tests. The software could not override the internal safety measures of the hearing aid. The maximum level of the pure tones delivered was 80 dB hearing level (HL), where HL was referenced to sound levels in dB re 20 µPa obtained from Ref.^57^.

The Soundworld HD100 Hearing Aid is a printed circuit assembly powered by a rechargeable lithium-ion battery. The main processing unit of HD100 is an open − programmable DSP − based hybrid specifically designed for wireless, high − performance hearing aids. The PCB incorporates a low-power Bluetooth 4.0 radio and all necessary passive components for interfacing with transducers. The Digital Signal Processor (DSP) core processor supports wireless protocols and combines an open − programmable controller with hardware accelerators for audio coding and error correction support. A Filter Engine enables time-domain filtering and supports an ultra − low − delay audio path. The design allows for 16-channel compression, feedback cancellation, 16-channel noise reduction, and several environmental modes. Only proprietary software written and distributed by Soundworld and its affiliates can be installed on the HD100. “Source code” (both firmware and software) is solely controlled by Soundworld and is not distributed to end-users. The HD100 employs two MEMS microphones, allowing for directional detection of sound. Three pushbuttons allow to power the device on and off, modify the volume, and change the program options. The output consists of a Receiver-in-the-Canal (RIC) speaker employed in the user’s ear canal. The HD100 is housed in a weatherized, plastic enclosure and houses the tuned antenna inside.

### The otoTune software platform

The otoTune app provides a user interface to connect the phone's left and right HD100 hearing aids. Once the devices are connected, another app mode can be accessed, displaying a left and right button on the smartphone’s screen to collect feedback from the subject. Mathematical code was implemented to perform the large computations necessary to compute GP classification on the mobile device’s hardware. Once the evaluation was completed, the results were saved to the smartphone's memory with any personal identifying information removed. The software also provided a mode to view a list of the collected data files, which could then be securely and wirelessly exported to a personal computer to conduct data analysis. All data were transferred to an encrypted hard drive and removed from the smartphone at the study's end.

### Hearing assessment

The process to obtain a threshold estimate using GP classification can be summarized by the following steps:

1. A pure tone was played through the left or right hearing aid. It was labeled “audible” or “non-audible”, depending on the subject pressing a button on the user interface, indicating that a tone was heard on the corresponding side. The 3-tuple represented each trial (frequency, sound level (dB HL), and response label (1 = heard, 0 = not heard).
2. A list of pre-specified tones was played to generate an initial probabilistic estimate of the subject’s hearing threshold curve.
3. The probabilistic estimate was used to select the next tone determined by which area in the space of tones and frequency the algorithm was most uncertain about.
4. A tone at this most uncertain point was played, and the subject provided feedback on whether this tone was heard or not.
5. The probabilistic estimate was computed again to generate a new point that the algorithm was most uncertain about, and that tone was played in the subsequent presentation.
6. The process continued iteratively until additional tones only provided marginal new information based on the change in the uncertainty estimate. The model could be viewed as a binary classifier for any pair of possible tones, with a decision boundary in the frequency-intensity space interpreted as the hearing threshold curve.

### The Gaussian process classification

The testing procedure used a Gaussian process classification model for each ear to estimate the probability that a tone can be heard over the space of dB HL intensity and frequency values. The Gaussian Process is outlined in Eq. (1).

1 $$p\left( {\left. {\text{f}} \right|{\text{X}}} \right)\, = \,N\left( {\left. {\text{f}} \right|\mu {,}\,{\text{K}}} \right),$$

where f is a function*,* X is the design matrix*,* $\mathcal{N}$ *p*(f|*μ,*K) is (the function f has a) Gaussian distribution with mean vector *μ* and covariance matrix K*, μ* is the mean function, and K is the covariance matrix.

The GP requires selecting a covariance function or covariance kernel; a squared-exponential covariance kernel was used in this application, shown in Eq. (2).

2 $$\kappa \left( {{\text{x}}_{i} ,\,{\text{x}}_{j} } \right)\, = \,\sigma_{f}^{2} \,\exp \left( { - \frac{1}{{2l^{2} }}\left( {{\text{x}}_{i} \, - \,{\text{x}}_{j} } \right)^{T} \,\left( {{\text{x}}_{i} \, - \,{\text{x}}_{j} } \right)} \right),$$

where *κ* (x_*i*_, x_*j*_) is the squared-exponential covariance function (kernel) evaluated at x_*i*_, x_*j*_, σ^2f^ is the variance of the (noise free) signal (σ determines the average distance of the function away from the mean), and *l* is the characteristic length scale, which determines the ripples in the function.

The standard GP model must be modified using a logistic function to perform classification to obtain a function whose output is between 0 and 1, which can be seen in Eq. (3)^54^.

3 $$f\left( x \right)\, = \,\frac{L}{{1 + e^{{ - k\left( {x - x_{0} } \right)}} }},$$

where *L* is the log-likelihood, $x$ is input, and *k* is a constant.

Pressing the corresponding left or right button during the tone delivery provided the user’s input. Each response was recorded and was 1 for pressing the correct button and 0 otherwise. With the patient’s prior responses, a continuous two-dimensional curve over the space of tones (dB HL x frequency) can be computed at any time during the test. The function range is a value between 0 and 1, interpreted as a probability estimate that the user can hear a tone at that intensity and frequency.

The GP also provides an uncertainty estimate for every possible probability estimate, where the uncertainty estimate curve has an identical domain as the probability estimate curve. This uncertainty estimate serves to determine the next tone and intensity. The selection is accomplished by choosing the point on the uncertainty estimate curve over the tones' space with the highest value. It is the tone that the probability model is most uncertain about and will be the tone that is played next, therefore maximizing the amount of information obtained. Once this next tone is played, its response is recorded as heard or unheard and added to the test dataset.

Another Gaussian process is then computed to obtain a new probability and uncertainty estimate curve for which a new tone will be selected. The process is repeated iteratively until approximately 35 responses per ear are collected. A final output curve for the left and right ear is obtained from the GP uncertainty curve given the complete dataset by selecting the maximum value from the uncertainty estimate curve across the entire range of frequencies. The algorithm is based on a probabilistic model, viewed as a two-dimensional binary classifier specified by a GP. The GP outputs a continuous estimate; therefore, a smooth function of frequency is obtained.

### Study participants and study design

The study was a single-center study. The criteria to include the patient in the study were: (1) the study participant is 21 years or older, (2) the study participant has English language proficiency, (3) the study participant can follow written instructions in English, (4) the study participant had an audiogram in the past six months, including the day of the patient’s visit to the clinic. All study participants with or without hearing loss were eligible if they did not have a severe-to-profound hearing impairment and had none of the exclusion criteria outlined below.

The criteria to exclude the patient in the study were (1) the study participant was not able to consent, (2) for the study participant, English is a second language, (3) the study participant was legally blind (central visual acuity of 20/200 or less in the better eye with best possible correction and a visual field of 20 degrees or less, (3) the study participant has a severe-to-profound hearing loss (> 70 dB HL), (4) the study participant cannot understand the written and verbal instructions given by the study software or by the study coordinator.

The study investigator brought the patient into a quiet examination room. Informed consent documentation was provided to the participant. The study responsible investigator explained the study participant answered questions from the patient regarding the study. Written consent to participate in the research projects was sought and obtained directly from the participants. Documentation consisted of a signed statement of informed consent approved by the Institutional Review Board (IRB). Participants were informed of the tasks and time involved in the study. They were also told that their participation is strictly voluntary and that choosing not to participate would not harm their relationship with their clinical care providers. They were also informed that if they decide to participate, they can withdraw from the research project at any time, for any reason, and without penalty. Participants were given a signed copy of their consent form. A four-digit random number was assigned to each patient.

All written and recorded data were de-identified. Upon entry into the study, participants received a randomized code number, used on all material generated by the research protocols and for data presentation in scientific and professional meetings. Computers used on the project were encrypted, password-protected, and access to files required two-factor authentication.

### Data analysis

The primary goal of this study was to determine if the audiogram values obtained from the standard clinical procedure were similar to those hearing thresholds collected using the self-guided *in-situ* test method.

The study has a cross-over design where each subject was used as their own control. M1 was used as described above and represented the control group. The experimental condition used M2. Statistical analysis included graphing the threshold curves obtained from M1 and M2 along with the difference curves, computing t-tests, running a MANOVA, and an analysis of credible intervals based on pure tone threshold differences at 250, 500, 750 1000, 1500, 2000, 3000, 4000, 6000, and 8000 Hz.

The outcome measure was the hearing sensitivity of each patient for a frequency range between 250 and 8000 Hz. The M1 procedure produced up to 11 discrete audiogram frequencies, whereas M2 produced a continuous function of frequency. Therefore, the difference between M1 and M2 were evaluated at the frequencies from M1 subtracted from M2 at those same frequencies to obtain differences.

The analysis included the following steps: (1) Graph the M1 standard audiometric data for all subjects, (2) plot the M2 experimental *in-situ* data for all subjects, (3) compare difference values at 250, 500, 750, 1000, 1500, 2000, 3000, 4000, 6000, and 8000 Hz for all subjects, (4) calculate the average threshold difference and corresponding standard deviations.

Since the study approach includes repeated measures (multiple frequencies for each evaluation method), a MANOVA was used. The Lilliefors test was applied to determine whether the sample data derived from the differences measured between M1 and M2 come from a distribution in the normal family. A credible interval analysis of the difference values across frequency. The power of the study was calculated.

The sample size for the study was determined before the start of the study using MATLAB-2018b, MATLAB-2019a (Mathworks, Natick, MA, USA), G*Power (RRID:SCR_013726), and IgorPro 8 (WaveMetrics Inc, Lake Oswego). After plotting and visually inspecting the plots, the actual power was calculated after the completion of the study with the same software. The a priori calculations for the sample size and power assuming a medium effect size (0.47) and an alpha of 0.05 suggested a target enrollment of 55 participants.

### Supplementary Information

Supplementary Information.

## Data Availability

All data analyzed have been shown in the paper. The raw data are available upon request from the corresponding author.
