# Supplementary material for: In-situ hearing threshold estimation using Gaussian process classification
Source: Sci Rep. 2023 Sep 6;13:14667. doi: 10.1038/s41598-023-40495-w (PMC10482858; doi:10.1038/s41598-023-40495-w)
Supplement: Supplementary file 1 — Supplementary Information. [file 41598_2023_40495_MOESM1_ESM.docx]

**Supplemental Materials**

***In-situ* Hearing Threshold Estimation using Gaussian Process Classification**

Christopher Boven^1^, Reagan Roberts^1^, Jeff Biggus^1^, Malini Patel^2^, Akihiro J. Matsuoka^2-6^, Claus-Peter Richter^3-5,7^

^1^ Soundwave Hearing, LLC; 619 Enterprise Drive #205; Oakbrook, Illinois 60523, United States of America.

^2^ Northwestern Medical Group, 675 N. St. Clair, Suite 15-200, Chicago, Illinois 60611, United States of America.

^3^ Department of Otolaryngology, Northwestern University, 320 E. Superior Street, Chicago, Illinois, 60611, United States of America.

^4^ Roxelyn and Richard Pepper Department of Communication Sciences and Disorders, School of Communication, Northwestern University, Evanston, IL 60201, United States of America.

^5^ The Hugh Knowles Center for Clinical and Basic Science in Hearing and its Disorders, Evanston, IL 60201, United States of America.

^6^ Center for Advanced Regenerative Engineering, Evanston, IL 60201, United States of America.

**^7^** Department of Biomedical Engineering, Northwestern University, 320 E. Superior Street, Chicago, Illinois, 60611, United States of America.


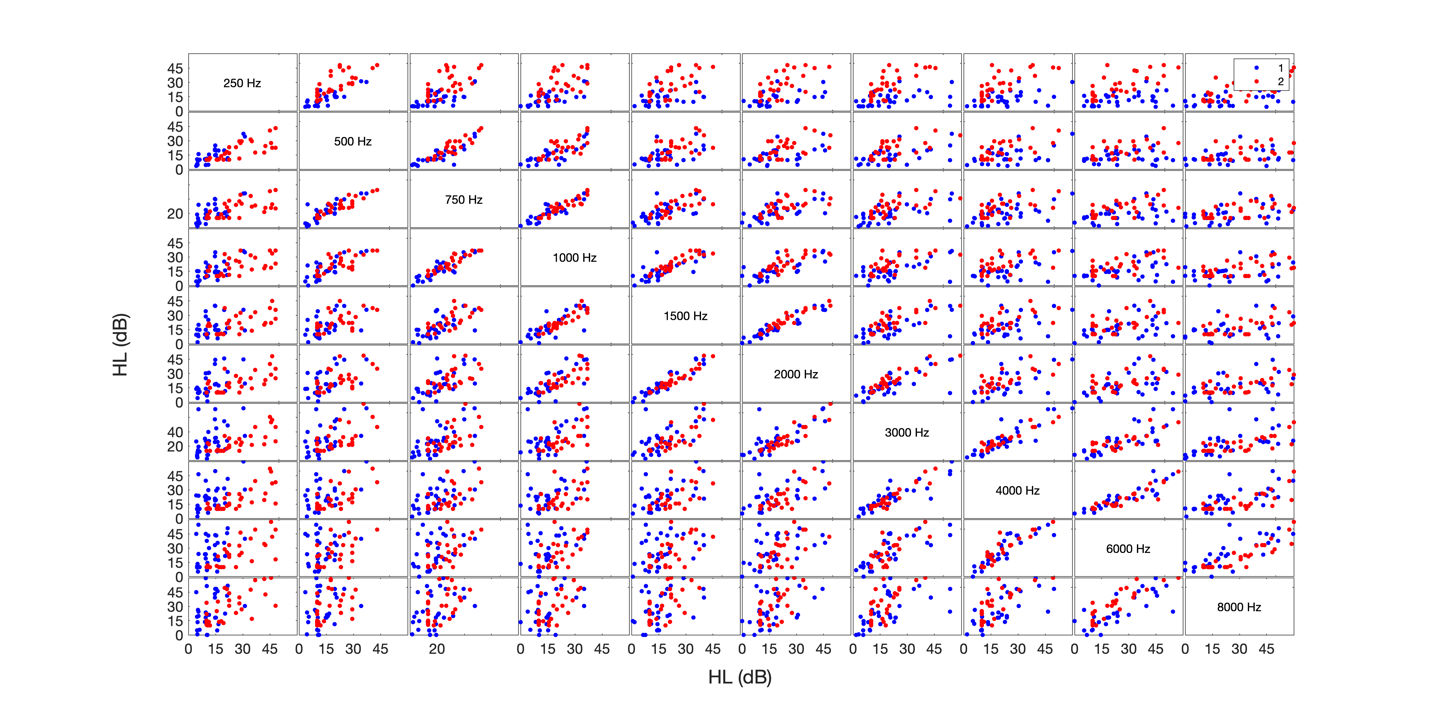


**SFigure 1.** The matrix plot shows the dependence among the repeated measures for each method for the right ears. Columns 1 and 2 and rows 1 and 2 show that the responses at 250 Hz and 500 Hz are more different between M1 and M2.


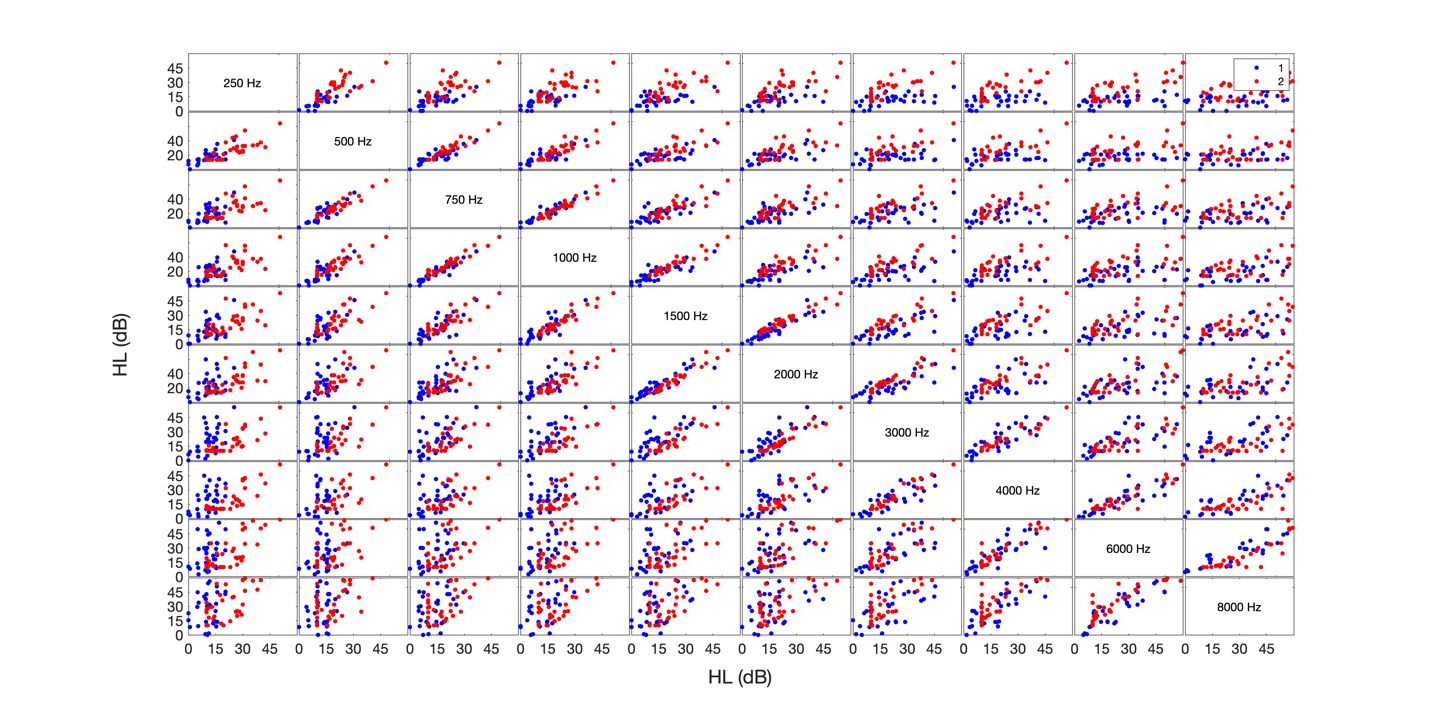


**SFigure 2.** The matrix plot shows the dependence among the repeated measures for each method for the left ears. Columns 1 and 2 and rows 1 and 2 show that the responses at 250 Hz and 500 Hz are more different between M1 and M2.


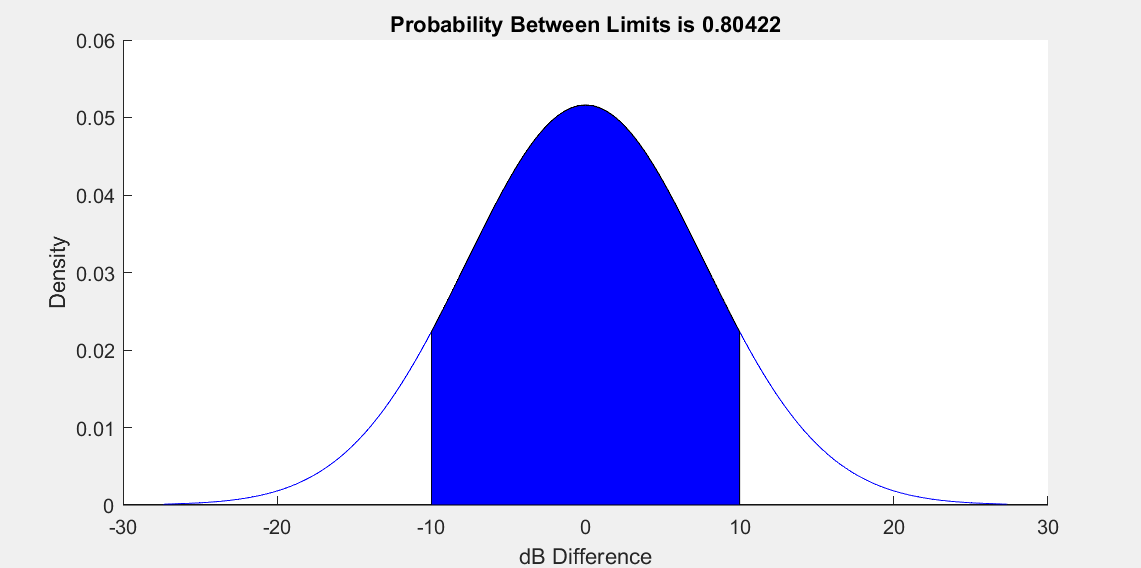


**SFigure 3.** A credible interval was computed for the difference between M1 and M2 in the left ear at 1 kHz, given an interval of +/- 10 dB with the mean difference set to zero. 80% of the probability mass is within the selected interval in this example.

|  | 250 Hz | 500 Hz | 750 Hz | 1000 Hz | 1500 Hz | 2000 Hz | 3000 Hz | 4000 Hz | 6000 Hz | 8000 Hz |
| --- | --- | --- | --- | --- | --- | --- | --- | --- | --- | --- |
| Left Ear CI | **0.69** | **0.76** | **0.78** | **0.80** | **0.75** | **0.71** | **0.71** | **0.80** | **0.64** | **0.56** |
| Right Ear CI | **0.53** | **0.68** | **0.69** | **0.69** | **0.80** | **0.75** | **0.66** | **0.62** | **0.56** | **0.49** |

**STable 1.** Credible intervals were computed for each frequency where each sample was a difference value between M1 and M2. 10 different frequencies were analyzed ranging from 250 Hz to 8000 Hz. The values predict the proportion of future samples that may fall within the acceptable region. CI: confidential interval.
